# Horticultural Activity: Its Contribution to Stress Recovery and Wellbeing for Children

**DOI:** 10.3390/ijerph17041229

**Published:** 2020-02-14

**Authors:** Yuhan Shao, Mohamed Elsadek, Binyi Liu

**Affiliations:** 1 Department of Landscape Architecture, College of Architecture and Urban Planning, Tongji University, Shanghai 200092, China; shaoyuhan@tongji.edu.cn (Y.S.); 89098@tongji.edu.cn (B.L.); 2 Department of Horticulture, Faculty of Agriculture, Suez Canal University, Ismailia 41522, Egypt

**Keywords:** children, relaxation, horticulture activity, horticulture therapy, heart rate variability, wellbeing

## Abstract

In recent years, children’s use of mobile phones has grown rapidly, which might lead to an increase in mental stress and negatively affect their health. Despite increasing evidence that horticultural activity can provide significant health benefits, few scientific evidence-based studies are currently available regarding these benefits to children’s health and wellbeing in schools. Therefore, this study aims to determine the potential benefits of horticultural activity for children from both psychological and physiological perspectives. Twenty-six elementary school students (mean age, 8.12 ± 0.21 years) were asked to perform a plant-related task and a mobile game task for 5 min. During both tasks, physiological sensors were used to measure the participants’ heart rate variability, skin conductance, and skin temperature. Additionally, the participants’ emotional responses were assessed using semantic differential and State–Trait Anxiety Inventory tests immediately after each task. Results revealed that, compared with the mobile game task, participants’ health statuses were positively correlated with the horticultural task, including a considerable decrease in skin conductance and sympathetic nervous activity, together with a marginal increase in parasympathetic nervous activity. Such responses suggested that horticultural activity increased relaxation and decreased feelings of stress. Furthermore, the horticultural activity was associated with a substantial increment in comfort, naturalness, relaxation, and cheerfulness feelings, as well as a significant reduction in depression and a reduction in total anxiety levels. Given these positive benefits, horticultural activity may provide a great contribution to children’s healthy life at school, prompt psychological relaxation and minimize mental stress relative to smartphone games.

## 1. Introduction

The World Health Organization (WHO) (2006) defined health as “a state of complete physical, mental and social wellbeing and not merely the absence of disease or infirmity” [1]. Soga and collogues defined “health” in a wide meaning, including physiological and psychological wellbeing [2]. Importantly, individuals’ modern-day lifestyle, which includes long hours indoors and unconscious isolation from nature, can cause serious problems for individuals’ health and wellbeing [3]. Most individuals’ mental health problems (such as depression, anxiety, stress, anger, and sadness) are attributed to their limited exposure to natural environments [4]. 

Less exposure to nature and green spaces can increase the vulnerability of individuals to the effects of stressful life events and environmental stressors that influence physiological and psychological health [5]. Nature in cities plays an important role in promoting a healthy society [6]. The theories of Kaplan [7] and Ulrich [8] support the idea that rehabilitation from attentional fatigue and stress is effectively facilitated by natural environments. There is growing evidence that direct exposure to natural environments provides a wide range of health benefits [9,10,11]. The fact that daily contact with nature can improve individuals’ health and be used as a form of preventive medicine is increasingly being recognized [6]. These findings particularly support the argument that contact with nature, plants and horticultural activities can improve human health and wellbeing, giving support to both Attention Restoration Theory and Stress Recovery Theory. Attention Restoration Theory (ART) [7] suggests that exposure to nature can help us improve our focus and ability to concentrate. Stress Reduction Theory (SRT) [8] proposes that exposure to nature may have a direct restorative effect on cognition and may reduce stress.

A recent study showed that children spend more than 7 hours a day engaged in different media activities (e.g., smartphones, Internet and TV)—more time than they spend in studying or sleeping, which leads to increased psycho-physiological stress [12]. Additionally, surveys conducted in the USA reported that about 1 of every 4 children will experience mental stress and that about 1 in 10 will have a serious emotional disturbance [13]. Such psycho-physiological stress may lead to significant reductions in cognitive performance. Children, however, can recover from stress, retrieve cognitive efficiency and promote their health by taking advantage of the benefits resulting from exposure to nature [5].

Horticultural activity is one of the most common ways to interact with nature and is enjoyed in many countries as a popular pastime [2]. In mental health, activities relating to nature (e.g., horticulture and gardening) offer opportunities to strengthen various health and wellbeing facets, foster positive interpersonal relationships that improve social inclusion, and encourage mental illness destigmatization [14]. Increasing focus has been given to the beneficial impacts of nature-related activities, such as gardening or horticultural activities, which are regarded as a promoter of health and wellbeing [15]. Scientists and health practitioners are increasingly aware of the potential health benefits resulting from gardening practices [16,17]. Earlier studies have demonstrated that gardening improves individual life satisfaction, wellbeing, positive emotions, community social work, and cognitive function [18,19]. A decline in symptoms of stress, anger, depression and anxiety have also been documented [18,20]. Consequently, involvement in gardening has become more widely recognized as a cost-effective health treatment [16] as well as a treatment or occupational therapy for people with psychological health problems, called “horticultural therapy” [21].

Previous studies have shown the physiological and psychological benefits that occur in adults after horticultural and gardening activities [2,22]. Horticultural activities have proven to be excellent personal and community activities that promote and enhance the wellbeing of elderly people [23]. Despite these positive impacts, surprisingly, to date, little physiological and psychological evidence is available on the positive impacts of horticultural activity on children’s wellbeing. More comprehensive and convincing evidence is still needed. Louv’s study [24] concluded that a decline in interaction with nature has contributed to a number of health and behavioral problems, especially for children, which can, in short, constitute a “nature-deficit disorder”. Hence, this research focused on the benefits of horticultural activity on children’s health and wellbeing. 

Therefore, the objective of this study is to investigate the psychological and physiological relaxation impacts of horticultural activity from a multi-dimensional perspective by recording heart rate variability (HRV), skin conductance (SC), skin temperature (SKT) and emotional responses in elementary students. Scientific analysis regarding the recorded data was carried out to provide rigorous evidence in support of the benefits of horticultural activity on children’s wellbeing.

## 2. Materials and Methods

Ethical approval for the present study was obtained from the Ethics Committee of Tongji University (2019tjdx283). The informed written consent of the parents and/or guardians as well as the verbal consent of the students prior to participation were provided. The experiment was conducted between 20 and 25 December 2018.

### 2.1. Participants 

Twenty-six elementary school students comprising 15 males and 11 females at the age of 8.12 ± 0.21 (mean ± SE) were recruited for this study. Experiment volunteer students were recruited using two different methods: (1) an e-mail detailing the study procedures was sent to the school director; (2) the researchers of the present study asked the students in person if they would like to participate. Selected participants obeyed the following criteria: no pre-existing physical and emotional disabilities that could influence physiological outcomes, no pre-existing heart-related condition and skin condition and no allergy to electrodes. The procedure of the experiment was fully explained to the participants prior to the beginning of the experiment.

### 2.2. Study Protocol

To investigate the participants’ physiological and psychological responses, two tasks were introduced: first, stem cuttings were prepared from a real plant (one of the most common methods of the asexual propagation of plants) and cultivated in pots filled with soil, which was performed as a horticultural activity. Second, participants played a gardening game that simulated a realistic environment in which children can grow seeds and plants using smartphones as the comparison. Before the experiments, the author taught each participant the fine motor skills required, such as how to prepare cuttings (cutting with scissors) and how to cultivate them easily (planting) so that they could work more comfortably. The choice of the plant for cuttings was based on its availability in the area of the experiment. The activities took place in a quiet room in which the students rested after the study day. The room temperature was set at 21 °C ± 0.7 °C, mean ± SE; the relative humidity remained at 58.2% ± 2.5%; and the illuminance was 2000 lux. 

**Figure 1:**
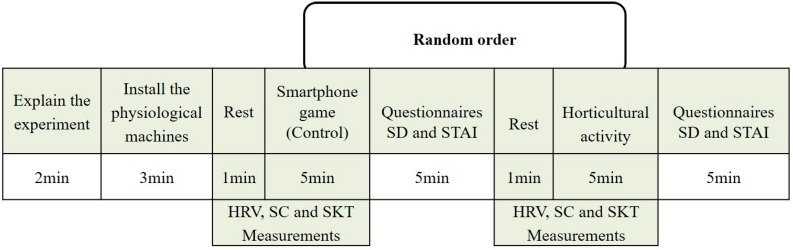
Study protocol. SD: semantic differential questionnaire; STAI: State–Trait Anxiety Inventory; HRV: heart rate variability; SC: skin conductance: SKT: skin temperature.

To ensure that participants required relaxation, the experiment was conducted after a school day. The time of the study was coordinated with classroom supervisors so that the experimental days fitted students’ schedules. Before starting the experiment, the process of the experiment and how to use the instruments were clearly introduced to the participants. The experiment design was within-subject, and both experimental conditions were experienced by each participant in a random sequence. Each participant was invited to sit on a chair while fitting the ErgoLAB sensors (Kingfar Technology Co. Ltd, Beijing, China) for HRV, skin conductivity and skin temperature measurements. Participants were then told to rest for 60 s to adapt their mood to the experimental environment and then asked to perform their assigned tasks of either a horticultural task or a smartphone game task for 5 min. At the beginning of the experiment, HRV, SC, SKT were recorded and continuously recorded until the end of each task. After that, each participant was asked to rate their emotional reaction and the level of anxiety for each task using two self-reported questionnaires: a semantic differential questionnaire (SD) and the State–Trait Anxiety Inventory (STAI). The participants were asked to relax again for a duration of 60 s after the completion of each task. The overall duration of the experiment was 27 min per subject (Figure 1).

### 2.3. Physiological Measurements 

The ErgoLAB synchronization platform (Kingfar technology Co. LTD, Beijing, China) was used to measure HRV, SC, and SKT. The ErgoLAB platform includes a set of portable sensors and a computer-based digital platform that is linked to a wireless receiver. Previous studies have verified the validity of ErgoLAB [25,26]

#### 2.3.1. Heart Rate Variability

A recent review revealed that HRV is affected by stress and encouraged its use in assessing psychological stability and mental stress in an objective assessment [27]. HRV represents the sympathetic level (stress) or the feeling of parasympathetic (relaxation) activation [28]. HRV is a useful tool for measuring the sympathetic–parasympathetic balance of the autonomic nervous system [29]. A power spectral analysis of the beat-to-beat variations of heart rate has become commonly used to measure cardiac autonomic regulation [30]. This analysis divides the overall variation (the “power”) of a continuous series of beats into its frequency components, usually defining two major peaks: a low-frequency band (LF) at 0.04–0.15 Hz (which is often thought to have a dominant sympathetic component) and a high-frequency band (HF) at 0.15–0.4 Hz (which represents cardiac parasympathetic nerve activity) [31]. In addition, the ratio of LF and HF (LF/HF) was determined as indicative of the sympathetic–parasympathetic nervous system activity ratio [31,32]. Lower HF power is correlated with stress, panic, anxiety, or worry [33]. A wireless wearable sensor, based on a photoplethysmogram (PPG) with a sampling frequency of 64 Hz, was attached to the participant’s earlobe to measure HRV values, LF, HF and the LF/HF ratio. Filters were applied before analyzing HRV results and then replaced with ectopic values to evaluate the frequency domain. Typical filters are low-pass de-noise, white de-noise, baseline de-noise, and band stop. The natural logarithm (Ln) was applied to frequency domain data before analysis to transform the original data into a normal distribution [34]. 

#### 2.3.2. Skin Conductance (SC)

Electrodermal activity, or sweating, has been long regarded as a measure associated with emotional arousal and stress. To measure the SC using the ErgoLAB platform, an electrodermal activity-based (EDA) wearable sensor with a 0–30 µs measurement range and 32 Hz sampling frequency was positioned on participants’ fingertips, which are areas of the body densely populated with eccrine sweat glands [35]. Two sensor electrodes were connected to two left-hand fingertips. In order to apply a time domain evaluation to the EDA signals, filtering procedures and image deconvolution analysis were carried out prior to the evaluation to remove noise [36]. In general, the SC response has been reported to be correlated with emotional responses (and especially high arousal responses) [37]. Such responses are controlled by the sympathetic system, which in turn activates the sudomotor nerve, causing the release of sweat from the sweat glands in the skin of the hand [38,39]. 

#### 2.3.3. Skin Temperature

The temperature of the skin is suggested to provide an indirect measure of activity in the sympathetic nervous system. A reduction in arousal results in an increase in vasodilation and blood flow to the peripheral areas of the body, demonstrated by an increase in skin temperature [35]. Previous work has shown that the temperature of the skin indicates the intensity of the stress [40]. A wireless skin temperature probe with a 10 °C to 60 °C measurement range, 0.1 °C precision, and 32 Hz sampling frequency was placed on the fingertip of the left hand to monitor temperature changes in the skin.

### 2.4. Psychological Measurements

Two self-reporting questionnaires were used during the experiment to evaluate the associated psychological reactions to each task.

#### 2.4.1. Semantic Differential Questionnaire (SD) 

Opposing adjectives were provided in the SD questionnaire: “comfortable to uncomfortable”; “natural to artificial”; and “relaxed to awakening” [41]. The question format was scored on a five-point scale (−2, −1, 0, 1 and 2) based on the emotional level. In this procedure, a higher score represented better emotional conditions.

#### 2.4.2. State–Trait Anxiety Inventory (STAI)

To assess the volatility of the anxiety level following both activities, the State–Trait Anxiety Inventory (STAI) investigated the children’s feeling “right now”, with 20 questions used to evaluate the participants’ corresponding feelings, such as tension, nervousness, and worry [42]; the higher the score, the more severe the anxiety [43].

### 2.5. Statistical Analysis

SPSS (Ver. 24; IBM Corporation, Armonk, NY, USA) was used to conduct a one-way ANOVA. Paired *t*-tests were used to compare physiological data, and for the psychological data, the Wilcoxon signed-rank test was used. A *p*-value of < 0.05 indicated statistical significance. All data were expressed as mean ± standard error.

## 3. Results

### 3.1. Physiological Measurements

#### 3.1.1. Heart Rate Variability (HRV)

**Figure 2:**
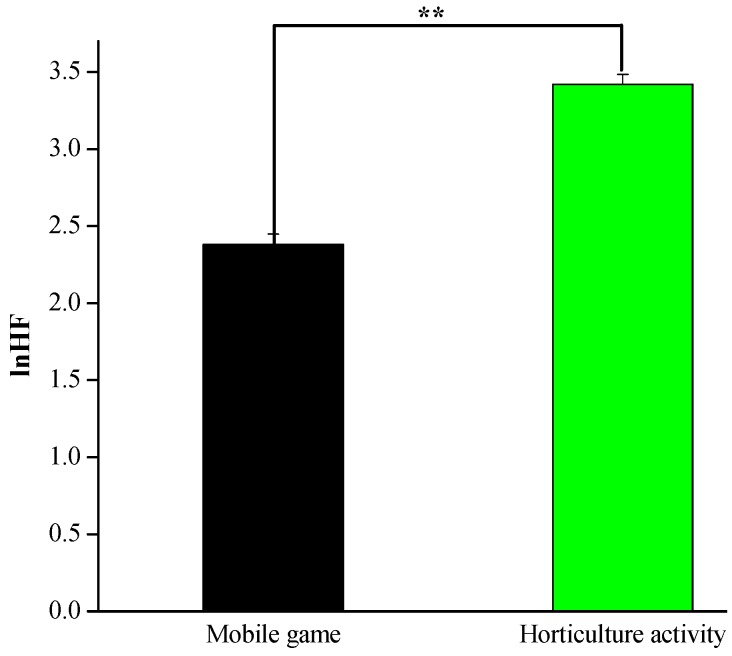
High-frequency band (lnHF) scores during the 5 min experimental period; *n* = 26, mean ± SE, ** *p* < 0.01 determined by the paired *t*-test.

**Figure 3:**
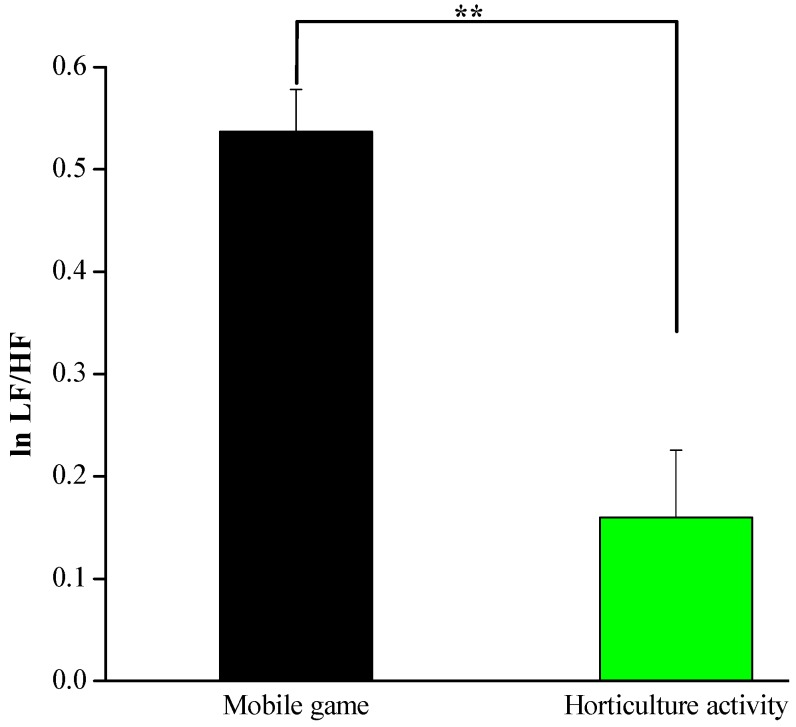
Ratio of low-frequency to high-frequency band (lnLF/HF) during the 5 min experimental period; *n* = 26, mean ± SE, ** *p* < 0.01 determined by the paired *t*-test.

The mean of the sympathetic and parasympathetic nervous activities while engaging in the horticultural activity and the mobile game activity, along with the associated test results for statistical significance, are presented in Figure 2 and Figure 3. The participants’ high-frequency band (lnHF), which represents parasympathetic nerve activity, significantly increased during the horticultural activity compared with those measured during the mobile game task (3.42 ± 0.06 vs. 2.37 ± 0.08, *p* < 0.01, Figure 2). Furthermore, as shown in Figure 3, the mean lnLF/HF, which represents sympathetic nerve activity, significantly decreased during the horticultural activity compared with that measured during the mobile game task (0.16 ± 0.07 vs. 0.54 ± 0.04, *p* < 0.01). This outcome suggests that the state of relaxation of the participants was associated with the horticultural activity rather than the mobile game task.

#### 3.1.2. Skin Conductance (SC)

**Figure 4:**
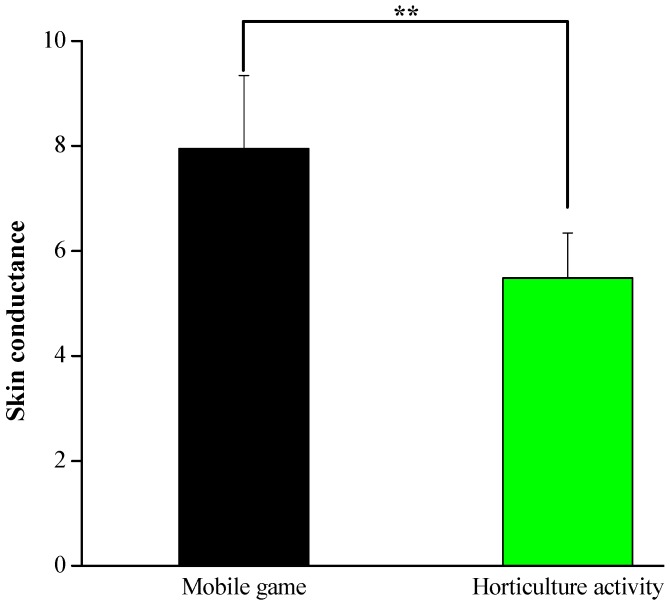
Mean of skin conductance during the 5 min experimental period; *n* = 26, mean ± SE, ** *p* < 0.01 determined by the paired *t*-test.

Figure 4 shows the mean of the participants’ skin conductance during the 5 min activity period. The participants presented lower SC averages during the horticultural task than during the mobile game task (5.48 ± 0.86 vs. 7.94 ± 1.40, *p* < 0.01). The results showed that the horticultural activity positively influenced the participants, as shown by noticeable declines in their skin conductance compared to the mobile game activity.

#### 3.1.3. Skin Temperature (SKT) 

**Figure 5:**
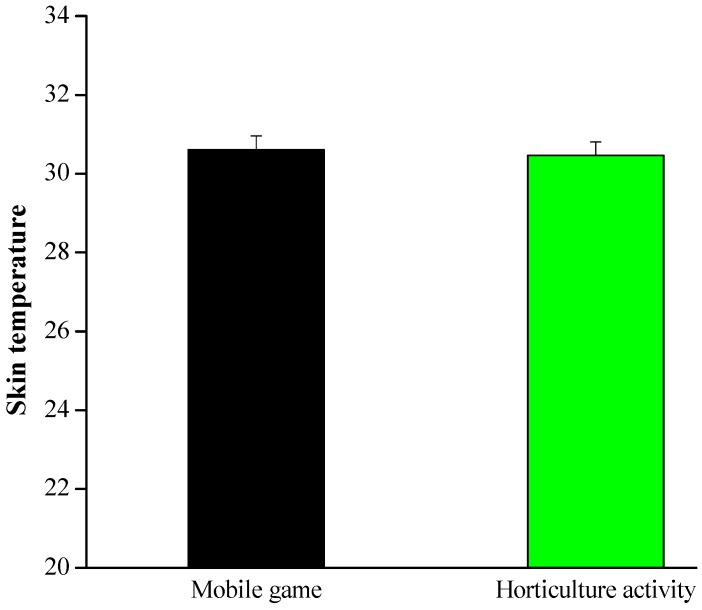
SKT during the 5 min experimental period; *n* = 26, mean ± SE, *p* > 0.1 determined by the paired *t*-test.

During the 5 min experiment period, with a sampling frequency of 16 Hz, the ErgoLAB platform’s wearable sensor was used to record the skin temperature of participants in the middle fingertip. Figure 5 shows that the two activities shared similar SKT mean values: horticultural activity, 30.47 ± 0.33 °C vs. mobile game, 30.60 ± 0.34 °C, *p* > 0.1.

### 3.2. Psychological Measurements

#### 3.2.1. Semantic Questionnaire (SD) 

**Figure 6:**
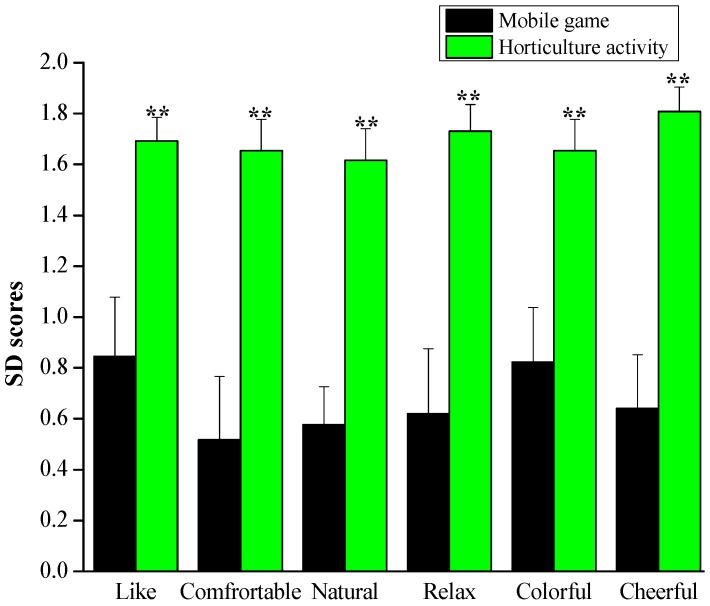
Comparison of the participants’ SD scores between horticultural and mobile game activities. *n* = 26, mean ± SE, ** *p* < 0.01 according to the Wilcoxon signed-rank test.

Semantic questionnaire data analysis revealed that emotions during the horticultural activity were different from those during the mobile game task. The participants felt more comfortable, relaxed, colorful and cheerful than after the mobile game task. Additionally, the participants expressed a strong preference for the horticultural task over the mobile task. There were significant differences between the two activities for the six feelings tested after the 5 min tasks (*p* < 0.001, Figure 6). Thus, engaging children in some horticultural activities may evoke more comfortable, relaxed, and positive feelings than playing games on phones.

#### 3.2.2. State–Trait Anxiety Inventory (STAI)

**Figure 7:**
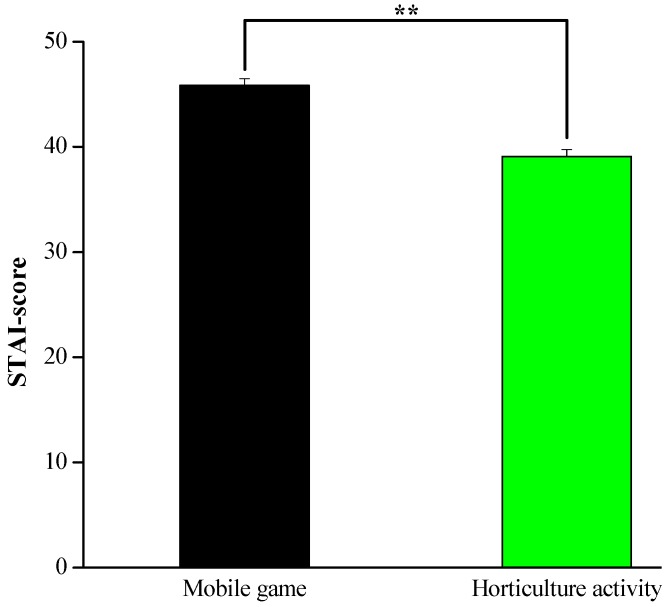
Comparison of participants’ State–Trait Anxiety Inventory (STAI) scores between the two activities. *n* = 26, mean ± standard error. ** *p* < 0.01, determined by the Wilcoxon signed-rank test.

The participants’ mean anxiety scores were lower after the horticultural task compared with those after the mobile game task (39.09 ± 0.64 vs. 45.85 ± 0.52; *p* < 0.01, Figure 7). This finding revealed the potential of horticultural activity to reduce anxiety levels.

## 4. Discussion

The objective of the present study was to investigate the health benefits and the stress-reducing impacts of 5 min of horticultural activity on elementary school students by measuring their physiological functioning (HRV, SC, and SKT) and psychological responses using SD and STAI from different assessment dimensions. Prior studies have shown the importance of horticultural activity for adult males [22], females [44] and elderly people [45]; however, very little is known about its positive impacts for children. 

Results showed that horticultural activity for 5 min resulted in lower LF power and higher HF powers than those obtained during the mobile game activity. Additionally, we found that the LF/HF ratio when the participants engaged in the horticultural activity was significantly lower than that obtained during the mobile game activity. It is interesting to note that horticultural activity significantly increased the children’s parasympathetic nerve activity, which stimulates relaxation, and significantly decreased the sympathetic nervous activity, which alleviates stress. In contrast, the mobile task increased the participant’s sympathetic nervous system activity. The results showed that horticultural activity could enhance children’s wellbeing by increasing relaxation and reducing stress compared with the mobile game activity. The lower LF/HF ratio represents parasympathetic superiority, as seen when people engage in tend-and-befriend behaviors [33]. Furthermore, prior studies reported that LF/HF was more likely to be increased at discomfort level [10,22]. These results reflect those of Lee and colleagues who found a significant stress reduction when the participants were involved in horticultural activity [22]. 

Skin conductance is an additional indicator of emotional state and physiological arousal and is a concept used to describe the electrical characteristics of the skin. Previous studies showed that the sympathetic autonomic nervous system, which controls the sweat glands in the skin, is influenced by an increase in physio-psychological arousal [46] and stress [47], which in turn increases the skin conductivity. The outcomes demonstrated that skin conductance was considerably lower when the children were engaged in the horticultural activity in comparison with the mobile game activity, indicating that they were more relaxed during the horticultural activity. The study by Ulrich showed that exposure to the natural environment has critical benefits for individuals’ heart rate and skin conductance [8]. These results suggest that horticultural activity can have positive impacts on human stress response through the suppression of sympathetic nervous system activity. These positive benefits may result from multiple natural stimuli acting on the five senses: touch, vision, listening, smell and taste [48]. Viewing plants has been documented to provide a variety of advantages including reduced anxiety and reduced stress [49,50,51,52]. In addition, Koga and Iwasaki [53] showed that touching plants induced physiological and psychological relaxation. Physiological and psychological stress can be reduced by touch, as the physical sensation of touch affects the cardiovascular system resulting in lower blood pressure and heart rate [54]. Unexpectedly, during the 5 min task period, there was no significant difference in mean SKT scores between the two activities. This may have been due to the experimental room temperature and humidity [35].

Regarding the results of the semantic differential questionnaire, subjects indicated that they felt more “comfort”, “relaxation”, and “cheerfulness” after participating in the horticulture activity compared to the mobile game activity. In words, the SD scores showed that horticultural activity positively affects the participants’ psychological states. Such results were consistent with Elsadek’s study, which reported that viewing plants has a strong positive correlation with positive emotions [55]. Additionally, transplanting activities led to better emotional states and lower stress levels than computer tasks [22]. On the other hand, the findings of the STAI scores also indicate that lower levels of negative emotions such as tension, anxiety, worry, confusion and unhappiness were felt when performing the horticultural activity compared to the mobile game task, offering additional support for the beneficial impacts of horticultural activity for human health. Together with other studies’ findings, the study results clarified that, when exposed to natural stimuli, participants are more likely to express lower levels of anxiety and negative emotions [56,57]. The self-reported questionnaires’ results for the participants suggest that horticultural activity has positive effects on mental stress and can reduce psychological stress compared with the mobile game task. 

It is worth emphasizing that the present study may support the beneficial physiological and psychological impacts of horticultural activity on children’s wellbeing via a scientific experiment. Participants tended to relax more during the horticultural activity due to their enhanced physiological and psychological levels, which is compatible with the observation of self-assessment that the participants felt much more relaxed and comfortable when engaging in horticultural activity. Interestingly, the response detected by the heart rate variability findings is substantially compatible with the results for skin conductance, and the emotional responses that horticultural activity provided were positive influences on the participants’ health and wellbeing. Two main theories that are useful in explaining the effect of horticultural activity on human wellbeing are Attention Restoration Theory [7] and Stress Reduction Theory [58]; both are based on the concept of biophilia—the idea that people have an innate need to be associated with the natural environment in which they have evolved. Although several studies have reported the positive impacts of horticultural activities [59,60,61], little attention has been given to children’s responses. Our experiment provides relevant data, from the perspective of relaxation, that can clarify the mechanism behind the health benefits of horticultural activities for children. Interestingly, our study suggests that the engagement of children in horticultural activities in school could reduce stress and promote their physiological and psychological relaxation.

Given the strengths and weaknesses of the present study, a notable strength is that the results are based on multi-dimensional measurements; the research combined different aspects of the physiological measurements (HRV, SC and SKT) with psychological aspects (SD and STAI). Furthermore, the lack of studies on the relaxation effects of horticultural activity for children underpins the significance of our study. This study was subject to some limitations, including that our focus group was limited to healthy children; in the future, more children with health problems should be examined to generalize the findings. Our sample size was small; future studies should recruit more participants. Furthermore, a comparison of children’s responses before/after the horticultural activity is needed, and we used only short-time measurement; theoretically, a long-term task is needed. Future research should also use more realistic and practical activities concerning the application of findings.

## 5. Conclusions

The present study provided scientific support for the physiological and psychological impacts of horticultural activity on elementary school students. Horticultural activity for 5 min enhanced children’s physiological relaxation by suppressing sympathetic nervous system activity and skin conductance compared with mobile use. Furthermore, the horticultural activity tended to relieve anxiety levels and bring several positive health benefits such as comfort, relaxation, cheerfulness and natural emotions for children. It was decisively shown that children could greatly benefit physiologically and psychologically from horticultural activity. In general, the results have important applications and great potential to be integrated as a daily program for students in schools.
